# Peptide–Carbon Nanotube Hybrids under Confinement: Structure and Stability from Atomistic Simulations

**DOI:** 10.1021/acsomega.5c12748

**Published:** 2026-02-11

**Authors:** Karinna Mendanha, Guilherme Colherinhas

**Affiliations:** Instituto de Física, Universidade Federal de Goiás, Goiânia 74690-900, GO, Brazil

## Abstract

The interaction between peptides and carbon nanotubes (CNTs) represents a promising route for developing biofunctional nanomaterials that couple structural flexibility to superior electronic performance. In this work, we investigate the structural and energetic behavior of A_6_D peptides confined inside a single-walled CNT using classical molecular dynamics simulations. The system consists of a 2 nm-radius CNT containing 35 A_6_D peptides and an equivalent number of counterions, fully solvated in water. Analyses of hydrogen-bond dynamics, Coulombic and van der Waals energies, and Ramachandran distributions reveal that peptide–solvent interactions dominate peptide–peptide aggregation, maintaining high flexibility within the confined environment. The alanine residues exhibit strong hydrophobic attraction to the CNT surface, while aspartic acid residues form extensive hydrogen bonds with water, resulting in a balanced solvation–stabilization regime. The confined peptides preferentially adopt α-helical conformations compatible with the cylindrical geometry of the nanotube, suggesting the potential formation of an internal peptide-membrane-like structure. These findings provide molecular-level insights into how electrostatic (peptide–peptide) and dispersion forces (peptide–peptide and peptide–CNT) govern organization and stability under nanoscale confinement. The results highlight the potential of peptide-coated CNTs as building blocks for bioelectronic interfaces, selective molecular transport systems, and controlled-release nanocarriers, bridging biomolecular self-assembly with advanced carbon nanotechnology.

## Graphical abstract

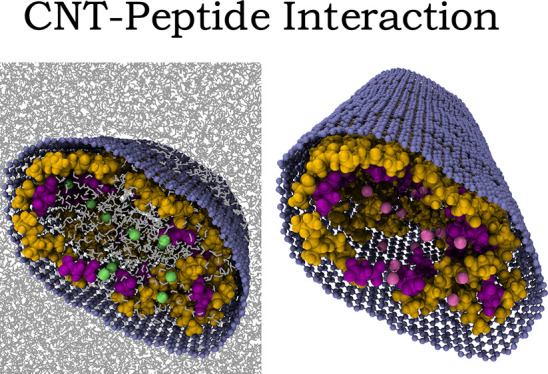

## 1 Introduction

The interaction between peptides and carbon nanotubes (CNTs) has emerged as a growing topic of interest in materials science and biotechnology owing to the potential to combine the exceptional electronic and mechanical properties of CNTs with the structural and functional versatility of peptides. Early studies in this field have explored how covalent and noncovalent linkages between peptides and CNTs can markedly alter the electronic, structural, and energetic characteristics of these nanostructures.^1^ Theoretical investigations based on density functional theory (DFT) have demonstrated that peptide hybridization modifies the dipole moment, electronic band gap, and stability of CNTs, emphasizing the determining role of bonding geometry.^1^ Such chemical modification provides new pathways to control the polarity and conductivity of nanomaterials, enabling the selective functionalization of carbon surfaces for electronic and biomedical applications. The growing interest in peptide–CNT conjugation has also been driven by the relevance of these systems in biocompatibility, molecular adsorption, and biomolecular transport.^2^ Molecular dynamics (MD) simulations have shown that functionalized CNTs enhance the adsorption affinity of therapeutic peptides and strengthen both hydrophobic and van der Waals interactions.^2^ Peptides enriched in aromatic residues (e.g., Trp and Tyr) display stronger affinity for CNT walls through π–π stacking interactions, a factor identified as critical for stabilizing noncovalent associations.^3,4^ This trend has been corroborated by experimental phage-display studies, which revealed histidine- and tryptophan-rich sequences with selective affinity toward CNTs, acting as “symmetric detergents” capable of dispersing nanotubes in a controlled manner.^5^ Therefore, the rational design of peptide sequences becomes essential to modulating both the strength and selectivity of adsorption on carbon surfaces.

Beyond their role as dispersants, peptides have also been employed as supramolecular organizers and hierarchical assembly agents in CNT-based hybrid materials. Ionic peptides such as EFK_8_ can self-assemble around nanotubes, forming hybrid hydrogels with enhanced mechanical strength and electrical conductivity.^6^ These systems exhibit improved biocompatibility and have been proposed for use in tissue engineering and electrochemical devices. Similarly, the self-assembly of peptide nanofibers on graphene oxide has been shown to promote biomimetic hydroxyapatite mineralization, yielding biocompatible nanocomposites that support cell growth and adhesion.^7^ The same design concept has been extended to heterochiral tripeptides (e.g., l-Leu-d-Phe-d-Phe), resulting in self-healing conductive hydrogels with high structural homogeneity.^8^ Collectively, these results consolidate the role of peptides as structural and functional mediators capable of promoting dispersion, cohesion, and self-repair in carbon-based hybrid systems.

In parallel, the understanding of peptide–CNT interactions under confinement and external stimuli has progressed considerably. MD and force-probe MD simulations have revealed that directional mechanical forces influence peptide motion and conformational dynamics inside nanotubes, although the differences between pulling and pushing regimes remain relatively small compared to the effects of sequence and loading rate.^9^ Other studies have investigated peptide encapsulation and release within CNTs, demonstrating that external electric fields can overcome potential barriers and enable the controlled release of bioactive molecules.^10^ These findings are particularly relevant for the design of CNT-based drug-delivery systems, where the nanotube diameter, charge, and length can be tuned to optimize the molecular transport. Furthermore, nanotube curvature and geometric structure (armchair vs zigzag) have been identified as key factors governing the maintenance of peptide secondary structures, with armchair and zigzag CNTs exhibiting distinct efficiencies in encapsulation and helical preservation.^11,12^

The peptide–CNT interface has also stimulated the development of experimental applications in biosensing and biomolecular delivery. Peptides functionalized on CNTs have been integrated into electrochemical sensors for apoptosis detection^13^ and universal DNA recognition,^14^ demonstrating the versatility of these conjugates for rapid and sensitive bioanalysis. More recently, CNT–polymer–peptide composites have achieved successful DNA delivery into plant mitochondria, with efficiencies up to 30-fold higher than conventional methods, while α-aminoisobutyric acid modifications have improved membrane permeability and gene expression.^15^ Complementarily, peptide-functionalized CNT chemiresistors have revealed that the nanotube density directly influences noise levels and sensitivity, providing valuable design guidelines for volatile organic compound (VOC) and gas detection devices.^16^ These advances demonstrate that peptide–CNT hybrid systems have evolved beyond theoretical interest to form robust and multifunctional experimental platform.

However, few current studies have reported whether confined peptides can self-organize into a stable internal tubular arrangement inside a CNT. Moreover, a deeper understanding of the interplay between electrostatic and dispersion forces in this environment requires further investigation. In this work, we address this gap by investigating whether peptides confined within CNTs can spontaneously self-organize into stable internal architectures under realistic confinement conditions. To this end, we studied a system composed of CNTs internally coated with A_6_D peptides, employing classical MD simulations to elucidate how nanoscale confinement affects the structural, energetic, and dynamic behavior of the confined peptides (see Figure 1). We selected A_6_D as a model peptide, because the peptide loading was designed to achieve a high internal density within the CNT cavity, ensuring extensive peptide–peptide and peptide–CNT interactions without imposing steric constraints that would suppress translational or conformational freedom. The CNT geometry was deliberately chosen with a radius of 2 nm and a length of 7.1225 nm, enabling the accommodation of a relatively large number of peptides while avoiding complete filling of the nanotube interior. This CNT length is sufficiently large to minimize end effects while remaining computationally tractable. As a result, free internal volume is preserved, allowing spontaneous peptide rearrangement and self-organization under confinement rather than enforcing a densely packed or artificially immobilized state.

**Figure 1:**
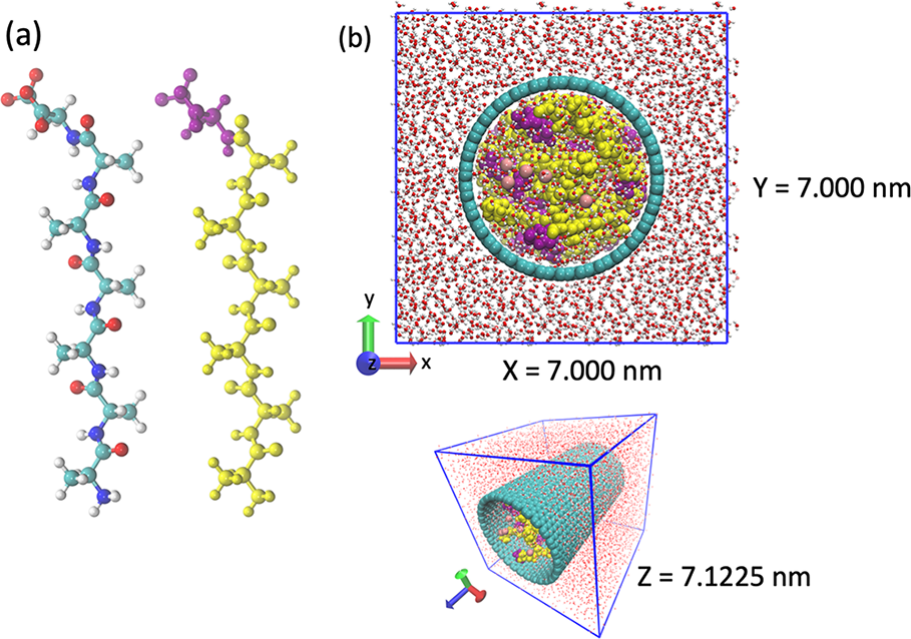
(a) A_6_D peptide: red = oxygen atoms; blue = nitrogen atoms; cyan = carbon atoms; white = hydrogen atoms; purple = aspartic acid (D); and yellow = alanine (A). (b) Initial configuration of the system, with the peptides and ions inside of the CNT structure.

The choice of a 2 nm radius (≈4 nm diameter) places the system in an intermediate confinement regime that is experimentally accessible in large-diameter single-walled and multiwalled CNTs, which have been widely employed in experimental and computational studies of confined water, ions, polymers, and biomolecules. In this regime, confinement is strong enough to induce interfacial ordering and collective effects yet not so restrictive as to impose single-file transport or highly constrained packing, which typically emerge in narrower nanotubes. Consequently, the model captures confinement-induced self-assembly, while avoiding artifacts associated with extreme geometric restriction. This balance is essential for probing self-assembly processes driven by confinement and interfacial interactions, which constitute the central focus of this work. The system was solvated to evaluate the influence of water on peptide–peptide and peptide-CNT interactions, thereby capturing the competition between solvation effects and attraction to the CNT walls. The analyses include hydrogen-bond statistics, Coulombic and van der Waals energy components, and Ramachandran plots, aiming to clarify the emergence of ordered structures under confinement and the stability arising from the balance between solvation and attraction to the nanotube walls. To ensure that the observed behavior is not dependent on a specific initial arrangement, additional simulations starting from different initial peptide configurations, while maintaining the same physical characteristics and simulation protocol, were performed and yielded consistent results (see Supporting Information, Table S1).

This work seeks to determine whether confined peptides can self-organize into a stable internal tubular arrangement analogous to a peptide membrane coupled to the CNT. Such structures could open opportunities for bioelectronic interfaces, selective biomolecular transport, and nanocarrier-based drug-delivery systems. Moreover, understanding the interplay between electrostatic and dispersion forces in this confined environment may provide valuable insights for the rational design of internal peptide coatings in nanotubes, enabling fine control over conductivity, hydrophobicity, and biocompatibility for applications in nanofluidics, sensors, and controlled-release technologies. A systematic exploration of other CNT diameters, chiralities, and peptide loadings, while highly relevant, is beyond the scope of this study and will be addressed in future work building upon the physical framework established here.

## 2 Methodology

The A_6_D (6 alanine [Ala,A] and 1 aspartic Acid [Asp,D]) peptide was initially constructed using PyMOL,^17^ as illustrated in Figure 1. A single-walled CNT with a radius of 2 nm was subsequently generated using VMD.^18^ To obtain the complete system depicted in Figure 1, Packmol^19^ was employed to insert 35 A_6_D peptides and 35 counterions—the latter compensating for the negative charges of the aspartic acid residues—inside the CNT cavity. The number of peptides was chosen to completely fill the inner volume of the nanotube, ensuring close packing without inducing steric overlap and ensuring homogeneous spatial distribution without atomic overlap and corresponding to the maximum peptide density achievable under confinement conditions. The resulting configuration was then solvated in explicit water using GROMACS,^20,21^ as shown in Figure 1. The A_6_D peptide was selected as a minimal and experimentally relevant model system that combines conformational adaptability with a well-defined polarity. Although alanine-rich sequences exhibit an intrinsic propensity toward α-helical conformations according to empirical criteria, such peptides are also known to form membrane-like assemblies with β-sheet alignment depending on environmental conditions and intermolecular interactions. In the present simulations, no secondary structure was imposed: peptides were initially introduced in a β-sheet-like arrangement within the CNT, allowing conformational ordering to emerge naturally under confinement. The inclusion of a single aspartic acid residue at one terminus introduces a localized negative charge and directional polarity, a feature that has been experimentally associated with the formation of self-organized peptide membranes and nanostructures.^22,23^ This design enables the investigation of confinement-driven self-assembly while avoiding additional complexity arising from increased charge density or aromatic interactions present in alternative sequences. Importantly, both the A_6_D peptide and the CNT dimensions employed here are experimentally accessible, allowing the modeled system to be regarded as a physically realistic platform for exploring peptide-functionalized nanotubes and membrane-mimetic nanostructures with potential applications in nanomaterials and biointerfaces.

To assess the reproducibility and robustness of the observed self-assembly behavior, three independent MD simulations were performed on the same system. Each simulation was initiated from a distinct starting configuration generated with Packmol, differing in the initial spatial arrangement and orientation of the peptides inside the CNT, while preserving the same number of peptides, CNT geometry, solvent composition, force-field parameters, and simulation protocol. This approach ensures that the resulting structural and dynamical features are not biased by a specific initial peptide arrangement but instead reflect an intrinsic confinement-driven behavior. All parallel simulations yielded consistent qualitative and quantitative trends, as summarized in the Supporting Information (Table S1).

All MD simulations were performed using the CHARMM36 force field^24–27^ for peptides and “opls_145B” for CNT atoms,^28,29^ while water molecules were represented by the TIP3P model.^28,29^ Equilibration and production MD-runs were conducted under a semi-isotropic NPT ensemble, which allows independent pressure control (Parrinello–Rahman^30^) along and perpendicular to the nanotube axis. This setup prevents artificial compression effects and ensures the adequate accommodation of solvent molecules and peptides within the confined region. The equilibration stage lasted approximately 10 ns, followed by a 100 ns production simulation. Temperature was maintained at 300 K using the v-rescale thermostat,^31^ and pressure was controlled by the semi-isotropic Parrinello–Rahman barostat.^30^ Electrostatic interactions were computed with the particle mesh Ewald (PME) method^32^ using a real-space cutoff of 1.2 nm, while Lennard–Jones interactions were truncated at the same cutoff. The equations of motion were integrated with a 1 fs time step, and all bond constraints were handled using the LINCS algorithm.^33^ A total of 50,000 configurations from the equilibrated trajectory were used for statistical analysis (the time evolution of the potential energy, temperature, volume of simulation box, and number of hydrogen bonds are shown in Figure S1 in the Supporting Information). Simulations were performed using GROMACS 2023,^20,21^ and all visualizations and postprocessing analyses were carried out with VMD.^18^

The composition of the simulated system is summarized in Table 1. Structural and dynamic properties were evaluated by the MD trajectories. Hydrogen-bond (HB) dynamics—including peptide–peptide and peptide–water interactions—were quantified according to the Luzar–Chandler geometric criterion,^34–36^ considering an HB present when the donor–acceptor distance was *r* ≤ 3.5 Å and the donor–hydrogen–acceptor angle θ ≤ 30°. Nonbonded interaction energies, encompassing Coulombic and van der Waals contributions, were averaged over the equilibrated trajectory to assess the internal energetic stabilization of the confined system. Furthermore, the conformational sampling of the peptide backbone was examined through Ramachandran plots,^37^ derived from φ and ψ dihedral distributions, to evaluate secondary-structure preferences and the flexibility of the peptides within the CNT confinement.

**Table 1:** Composition of Membrane Structures Highlighting the Start Dimensions of the Simulation Boxes and the Total Number of Atoms and Particles in Each Simulation

		composition			
structure	final simulation box length (nm)	# peptide	# water	# K^+1^	CNT atoms
A_6_D	(6.81, 6.81, 7.08)	35	9134	35	3480

## 3 Results and Discussion

### 3.1 Hydrogen-Bond Dynamics and Statistics

Evaluating the interactions among peptides confined within the CNT is essential to determining whether they form organized aggregates capable of assembling into a peptide nanotube coupled to the CNT surface. The average number of hydrogen bonds (HBs) between peptides indicates the presence of stable interactions that favor aggregation. The corresponding ΔG values, estimated as implemented in GROMACS,^20,21,34,35^ provide insights into the relative thermodynamic stability of the formed hydrogen bonds and enable the identification of energetically favorable conformations. It is important to emphasize that this Δ*G* does not represent a rigorous solvation free energy but rather serves as an effective energetic descriptor suitable for comparative analysis across different configurations of the same system. In addition, HB lifetimes, analyzed using the Luzar–Chandler^34–36^ formalism, reveal the persistence of intermolecular interactions and the mobility of the peptides within the nanotube, indicating whether the aggregate remains structurally stable or undergoes significant dynamic rearrangements. Taken together, these analyses characterize the ability of peptides to form a tubular aggregate structure under CNT confinement, providing molecular-level insights into the stability and organization of the simulated system.

The results presented in Table 2 show that each peptide forms, on average, 2.17 hydrogen bonds with other peptides while engaging in 20.47 hydrogen bonds with water molecules. These values indicate that, although some peptide–peptide interactions occur, most hydrogen bonds are established with solvent molecules. This behavior suggests that the peptides remain well-solvated and relatively dispersed within the nanotube, exhibiting limited aggregation. The predominance of peptide–water bonding highlights the crucial role of the solvent in stabilizing the system and preserving peptide flexibility, enabling them to adapt to the nanotube confinement without forming large rigid aggregates. A residue-level analysis reveals marked differences between alanine and aspartic acid. Alanine residues form, on average, 1.64 hydrogen bonds with water, whereas aspartic acid residues form 10.61. These findings reflect the intrinsic chemical nature of each residue: alanine, being hydrophobic, establishes few but more persistent interactions with water, while the polar, negatively charged aspartic acid forms numerous hydrogen bonds with solvent molecules. Together, these data confirm that solvation dominates the interaction landscape, keeping the peptides widely dispersed and structurally flexible inside the nanotube.

**Table 2:** Average Number of Hydrogen Bonds (HBs), Δ*G* (kJ mol^–1^), and HB Lifetimes (ps) Obtained from the Luzar–Chandler Theory^34,35^

	# HB	Δ*G*	HB-lifetime
peptide–peptide	2.17 ± 0.19 per peptide	24.57	3247.15
peptide–water	20.47 ± 0.53 per peptide	14.34	52.87
alanine–water	1.64 ± 0.06 per alanine	15.15	72.67
aspartic acid–water	10.61 ± 0.34 per aspartic acid	13.54	37.88

The free-energy analysis of hydrogen bonds (Δ*G*) further demonstrates that peptide–solvent interactions are thermodynamically more favorable than peptide–peptide ones. Peptide–peptide hydrogen bonds exhibit Δ*G* = 24.57 kJ mol^–1^, indicating a limited contribution to overall stability, whereas peptide–solvent bonds are considerably stronger, with Δ*G* = 14.34 kJ mol^–1^ per peptide. Among individual residues, hydrogen bonds between aspartic acid and water are slightly more favorable than those involving alanine, although the former are more numerous and shorter-lived. Overall, solvation plays a central role in structural stabilization, while peptide–peptide interactions provide only a minor contribution. HB lifetimes (Table 2) reveal striking differences between peptide–peptide and peptide–solvent interactions. Bonds formed between peptides are far more persistent, indicating greater temporal stability, despite their low abundance. In contrast, peptide–solvent hydrogen bonds are highly transient, reflecting the dynamic exchange characteristic of solvated systems. Among residues, alanine–water interactions display longer lifetimes than those of aspartic acid, suggesting that although Asp forms more hydrogen bonds, each is individually weaker and less stable.

The overall balance between short-lived peptide–solvent interactions and the few but long-lived peptide–peptide hydrogen bonds suggests that the confined peptides organize into a semiordered, solvent-mediated network rather than forming a continuous hydrogen-bonded peptide tube. This intermediate regime indicates that confinement promotes local ordering without inducing full aggregation, consistent with a flexible peptide layer coating the inner CNT surface. The coexistence of strong solvation and persistent peptide–peptide hydrogen bonds reflects a dynamic equilibrium between dispersion in the solvent and transient adsorption onto the CNT wall. This balance ensures that the system remains structurally stable while retaining molecular mobility, a feature that may be advantageous for diffusion-driven processes inside confined carbon nanostructures. From a functional perspective, this balance between solvation-driven flexibility and hydrogen-bond persistence may be crucial for designing peptide–CNT hybrid systems with tunable internal environments. Such systems could facilitate selective molecular transport or reversible adsorption, providing a foundation for bioelectronic interfaces and nanocarrier applications.

### 3.2 Nonbonded Interaction Energies: Coulombic and van der Waals Contributions

The evaluation of nonbonded interaction energies, specifically the Coulombic (electrostatic) and van der Waals contributions, is fundamental to understanding the stability and structural behavior of the peptide system confined within the CNT. Coulombic interactions reflect the electrostatic forces between charged residues, such as aspartic acid, and nearby ions or water molecules, playing a crucial role in stabilizing polarized conformations and neutralizing local charges in the system. In contrast, van der Waals interactions describe the short-range attractive and repulsive effects that govern peptide packing and spatial organization within a confined nanotube environment. The combined analysis of these energy components enables the identification of regions of greater stabilization, the evaluation of the peptides’ tendency to aggregate, and the understanding of how confinement influences system dynamics and flexibility. Therefore, Coulombic and van der Waals interactions constitute key parameters for interpreting the molecular forces that dictate the structural and energetic organization of peptides inside the nanotube.

Figure 2a shows the Coulombic interaction energies within the system. Peptide–peptide interactions exhibit an average energy of −2711.21 kJ·mol^–1^ per peptide, indicating strongly attractive forces that play a central role in maintaining structural cohesion within the nanotube. Peptide–solvent interactions display an average energy of −897.70 kJ·mol^–1^ per peptide, which is also attractive though less intense, suggesting that the solvent contributes to system stabilization without inducing significant aggregation. Peptide–ion interactions show an average of −129.76 kJ·mol^–1^ per peptide, indicating moderate attraction relevant to charge balancing and overall stability. As expected, no Coulombic contribution is observed for peptide–CNT interactions owing to the electrically neutral nature of the nanotube. Overall, all electrostatic interactions are attractive, with peptide–peptide interactions being predominant, while peptide–solvent and peptide–ion interactions complementarily sustain system stability without introducing notable repulsion. Figure 2b presents the van der Waals interaction energies. Peptide–peptide interactions display an average of −56.29 kJ·mol^–1^ per peptide, representing an attractive contribution that promotes peptide cohesion. Peptide–water interactions show an average of −12.27 kJ·mol^–1^ per peptide, which is also attractive, although less intense, reflecting additional stabilization provided by the solvent. Peptide–ion interactions exhibit an average of +6.43 kJ·mol^–1^ per peptide, indicating a slightly repulsive nature with a minimal contribution to stabilization. Conversely, peptide–CNT interactions show a significantly attractive average of −107.56 kJ·mol^–1^ per peptide, demonstrating a strong stabilizing effect of the CNT on the confined peptides. Taken together, these van der Waals interactions emphasize the importance of CNT confinement and peptide–peptide cohesion in maintaining the structural organization and overall stability of the system.

**Figure 2:**
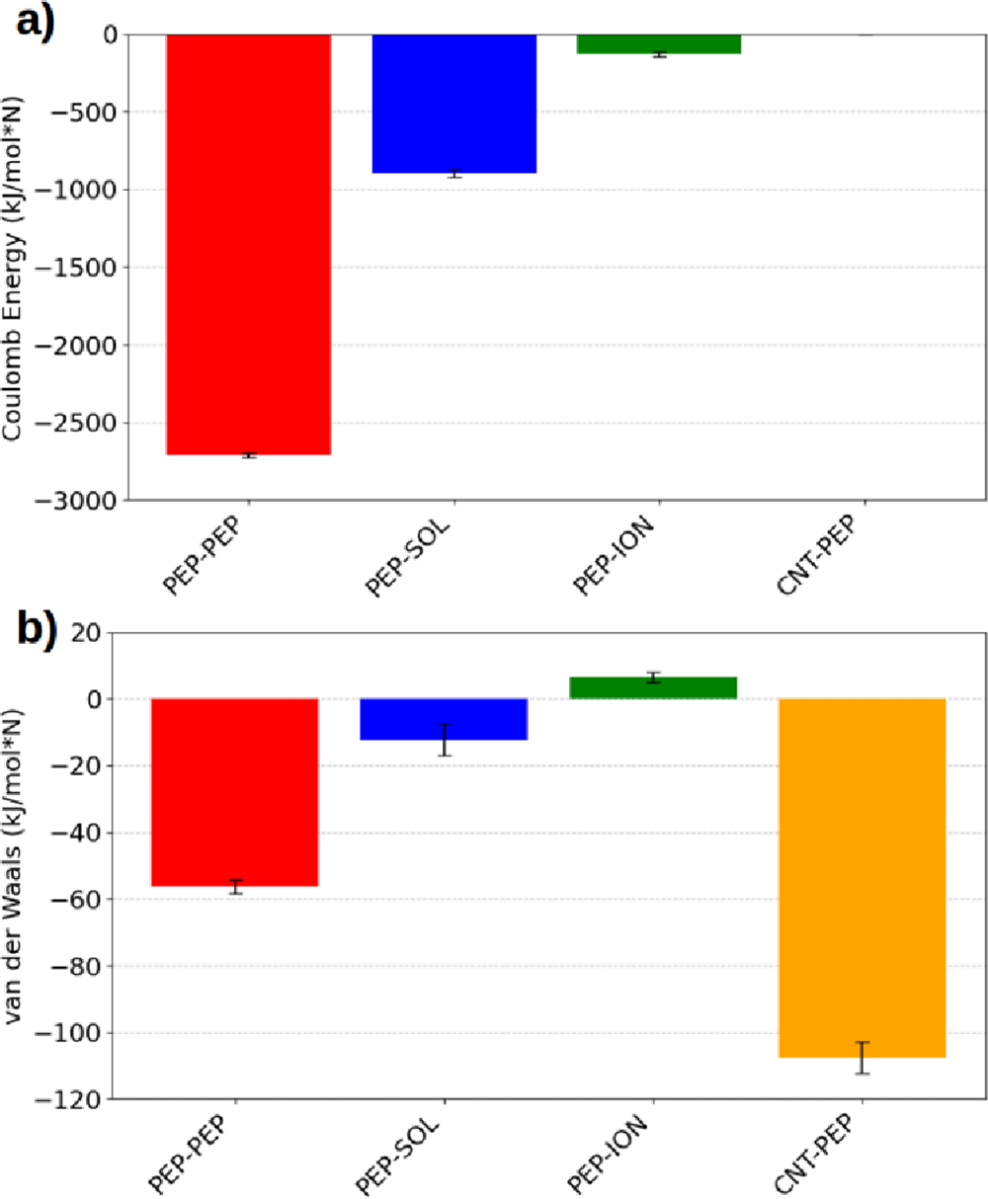
(a) Coulombic interaction energy and (b) van der Waals interaction energy between different components of the simulated system. The bars represent the average energetic contributions for peptide–peptide (pep-pep), peptide–water (pep-sol), peptide–ion (pep-ion), and CNT–peptide (CNT-pep) interactions. Error bars indicate the standard deviations computed over the equilibrated portion of the trajectory. These results highlight the predominance of attractive Coulombic interactions between peptides and the solvent and strong van der Waals coupling between the peptides and the CNT surface, reflecting the balance between solvation and adsorption inside the confined environment.

The balance between Coulombic and van der Waals interactions observed in this confined peptide–CNT system provides valuable insights into its potential functional applications. The coexistence of strong electrostatic cohesion among peptides and significant van der Waals stabilization at the CNT interface suggests that these hybrids could act as adaptive biofunctional coatings capable of maintaining structural integrity under dynamic conditions. Such a balance between internal peptide–peptide association and external CNT adsorption may be exploited to design nanocarriers with controlled-release properties, where the release rate of encapsulated biomolecules could be tuned by modulating the charge distribution or surface polarity. Furthermore, the pronounced van der Waals affinity between the peptides and the CNT walls indicates that peptide-functionalized nanotubes could serve as robust bioelectronic interfaces or molecular conduits, combining mechanical flexibility with enhanced electronic coupling. Overall, the energetic profile revealed here highlights a molecular mechanism by which confined peptide assemblies can be stabilized and functionally tailored for applications in biosensing, targeted delivery, and bioelectronic nanodevices.

Table 3 summarizes the Coulombic interactions between the peptide residues and the CNT, providing insights into the contribution of each residue to the overall electrostatic stabilization of the system. The strongest interaction was observed between Asp residues and the solvent with an average energy of −593.44 kJ·mol^–1^ per residue, highlighting the central role of charged residues in electrostatic stabilization through solvation. The Asp–ion interaction also contributed significantly, with an average energy of −127.10 kJ·mol^–1^ per residue, reflecting its importance in charge balancing and overall system neutrality. In contrast, Ala interactions were much weaker: the Ala–water interaction averaged – 50.63 kJ·mol^–1^ per residue, consistent with the nonpolar nature of alanine, while Ala–ion contributions were nearly negligible (−0.44 kJ·mol^–1^ per residue). As expected, no Coulombic contribution was observed for Ala–CNT or Asp–CNT pairs due to the electrical neutrality of the CNT. Overall, these results confirm that Asp residues dominate the electrostatic interactions within the system, whereas Ala residues play a secondary role in this regard.

**Table 3:** Average Coulomb (*E*_C_) and van der Waals (*E*_LJ_) Interaction Energies Per Residue (kJ mol^–1^·N^–1^) and corresponding Root-Mean-Square Deviations (rmsd) for Alanine and Aspartic Acid Residues Interacting with Water, Ions, and the CNT^a^

Coulomb energy (*E*_C_)	*E*_C_/*N* (kJ/mol)
alanine–water	–50.63 ± 2.66
aspartic acid–water	–593.44 ± 18.05
alanine–ions	–0.44 ± 0.75
aspartic acid–ions	–127.10 ± 15.50
alanine–CNT	0
aspartic acid–CNT	0
van der Waals energy (*E*_LJ_)	E_LJ_/N (kJ/mol)
alanine–water	–6.86 ± 0.58
aspartic acid–water	28.89 ± 3.00
alanine–ions	0.03 ± 0.08
aspartic acid–ions	6.22 ± 1.42
alanine–CNT	–17.35 ± 0.80
aspartic acid–CNT	–3.45 ± 0.46

a Negative values denote attractive interactions. The results highlight the strong electrostatic attraction of aspartic acid with water and ions in contrast with the predominantly hydrophobic behavior of alanine. The van der Waals contributions reveal moderate stabilization of alanine with the CNT surface and water molecules, while aspartic acid shows weaker dispersion interactions with the CNT, consistent with its polar and charged character. Together, these energetic trends emphasize the complementary roles of electrostatic and dispersion forces in governing the peptide–CNT confinement and interfacial organization. See Table S1 for more informations.

The van der Waals interactions, shown in Table 3, included both attractive and repulsive contributions. Among the attractive interactions, the most significant was Ala–CNT, with an average energy of −17.35 kJ·mol^–1^ per residue, followed by Ala–water, with −6.86 kJ·mol^–1^ per residue, indicating that nonpolar residues such as alanine exhibit strong affinity for the CNT confinement and, to a lesser extent, for the solvent. The Asp–CNT interaction was also attractive (−3.45 kJ·mol^–1^ per residue), suggesting a complementary role in stabilization near the CNT surface. On the other hand, notable repulsive contributions were identified for aspartic acid: Asp–water interactions averaged +28.89 kJ·mol^–1^ per residue, representing the primary destabilizing term, and Asp–ion interactions were also repulsive (+6.22 kJ·mol^–1^ per residue), albeit less intense. The Ala–ion interaction was nearly neutral (+0.03 kJ·mol^–1^ per residue) with no significant effect on system stability. In summary, the Ala–CNT attractive forces constitute the main stabilizing van der Waals contribution, whereas the Asp–water and Asp–ion repulsions represent destabilizing components that may influence peptide rearrangement within the confined environment.

Overall, these results reveal a cooperative balance between electrostatic and dispersive interactions that govern the structural organization of the confined peptides. Charged residues (Asp) ensure electrostatic neutrality and strong solvation, preventing excessive aggregation, while hydrophobic residues (Ala) mediate adhesion to the CNT surface through van der Waals attraction. This complementarity between polar and nonpolar contributions generates a stable yet flexible molecular assembly, which may be exploited in the design of peptide-coated nanotubes for controlled adsorption, selective transport, and bioelectronic interface applications. As illustrated in the cross-sectional view shown in Figure 3, the CNT exhibits small deviations from ideal cylindrical symmetry during the simulation. In the present work, the nanotube was modeled as a flexible structure, allowing its geometry to respond dynamically to internal stresses generated by the confined peptides and counterions. This behavior is consistent with previous studies, which demonstrate that flexible carbon-based walls can undergo moderate shape fluctuations leading to local variations in the effective confinement without qualitatively altering the dominant interfacial organization mechanisms.^38^ In this context, the observed deviation from perfect circularity reflects a physically realistic response of the CNT to heterogeneous internal loading rather than an artifact of the simulation protocol. Importantly, the peptide organization near the CNT wall reported here remains robust across the sampled trajectories, indicating that the main conclusions do not rely on the assumption of a rigid or perfectly cylindrical nanotube.

**Figure 3:**
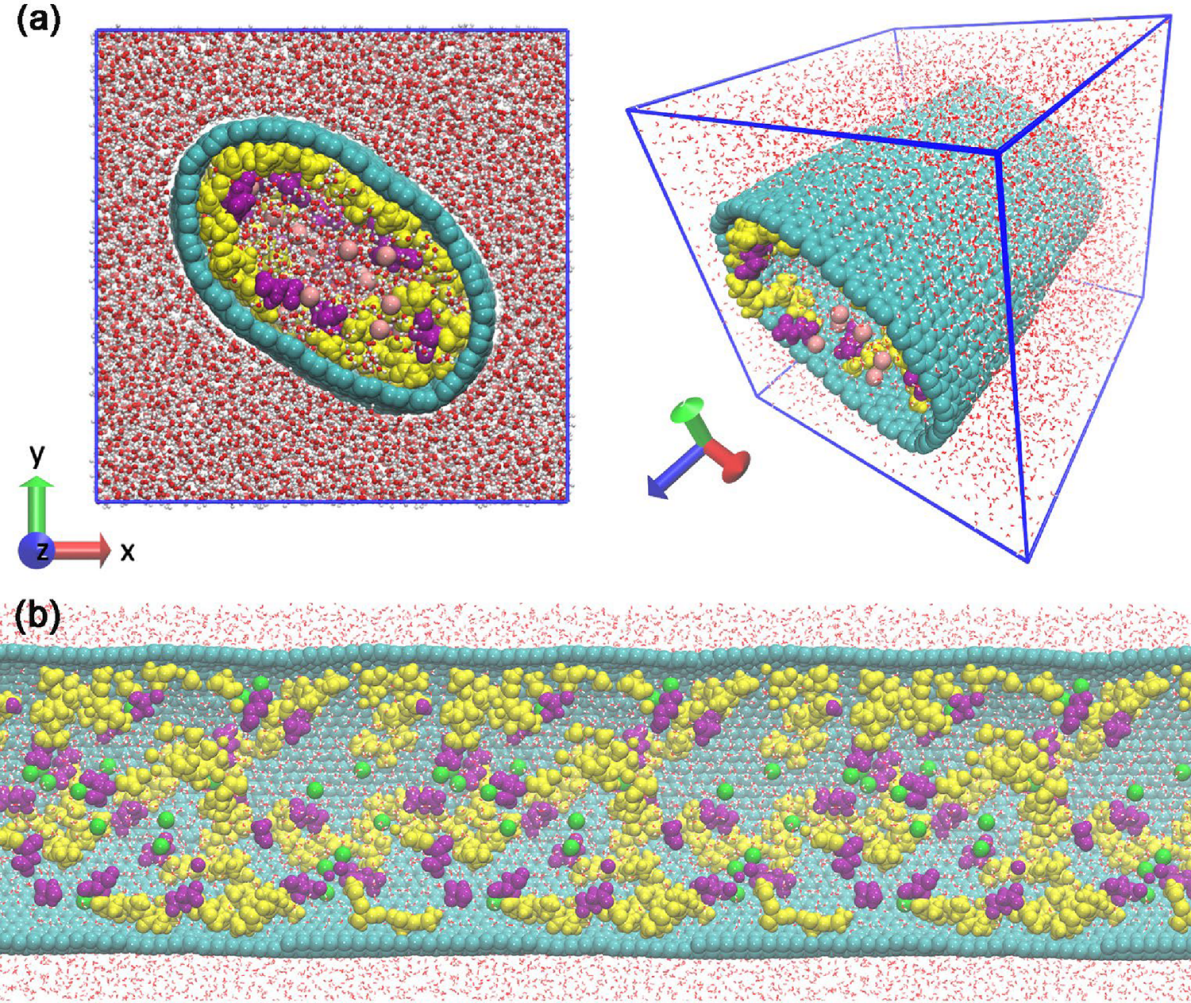
(a) Representative molecular final configurations of the confined A_6_D peptide system inside the CNT. Alanine (yellow) and aspartic acid (purple) residues along the CNT axis, evidencing the formation of a semiordered peptide layer coating the inner wall of the nanotube. (b) Representative molecular final configuration of the peptides distributed on the inner surface of the CNT with a longitudinal view (*Z*-axis). The color scheme emphasizes the preferential localization of hydrophobic alanine residues near the CNT surface and the positioning of polar aspartic acid residues toward the central, solvent-accessible region, reflecting the balance between hydrophobic confinement and electrostatic solvation that governs the structural organization of the confined peptides.

To further substantiate the role of CNT–peptide interactions in driving interfacial organization, radial distribution functions (RDFs) were computed between the peptides and the CNT, providing a quantitative description of peptide positioning relative to the nanotube inner surface (Figure 4). The RDF analysis reveals a pronounced accumulation of peptides near the CNT wall (*r* < 0.5 nm), with residue-resolved profiles showing that alanine residues exhibit the highest probability of close contact with the carbon surface (*r* < 0.75 nm). This behavior is consistent with the hydrophobic nature of alanine and its strong dispersive affinity for the CNT, and this directly corroborates the energetic analysis indicating dominant van der Waals contributions at the interface. By moving beyond visual inspection and global energy trends, these quantitative radial profiles demonstrate that CNT–peptide dispersive interactions are the primary driving force behind the formation of the semiordered peptide layer observed under confinement, thereby reinforcing the mechanistic interpretation proposed in this work.

**Figure 4:**
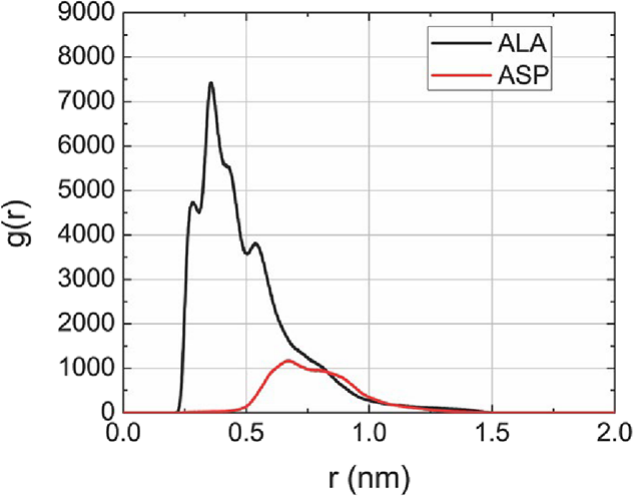
RDFs between peptide residues and CNT carbon atoms. The red line corresponds to alanine residues (A) and the black line to aspartic acid residues (D), showing a higher probability of alanine residues near the CNT inner surface.

### 3.3 Ramachandran Plots

Figure 3 illustrates the structural organization of the confined A_6_D peptides inside the CNT, revealing the formation of a continuous peptide coating along the inner CNT wall. To assess the influence of nanoscale confinement on the peptide structure, the conformational distributions of the φ (phi) and ψ (psi) dihedral angles were analyzed through Ramachandran plots (Figure 5). This approach provides a detailed view of the conformational space most frequently explored by the peptide chains, offering valuable insights into their stability, flexibility, and secondary-structure preferences under confinement.

**Figure 5:**
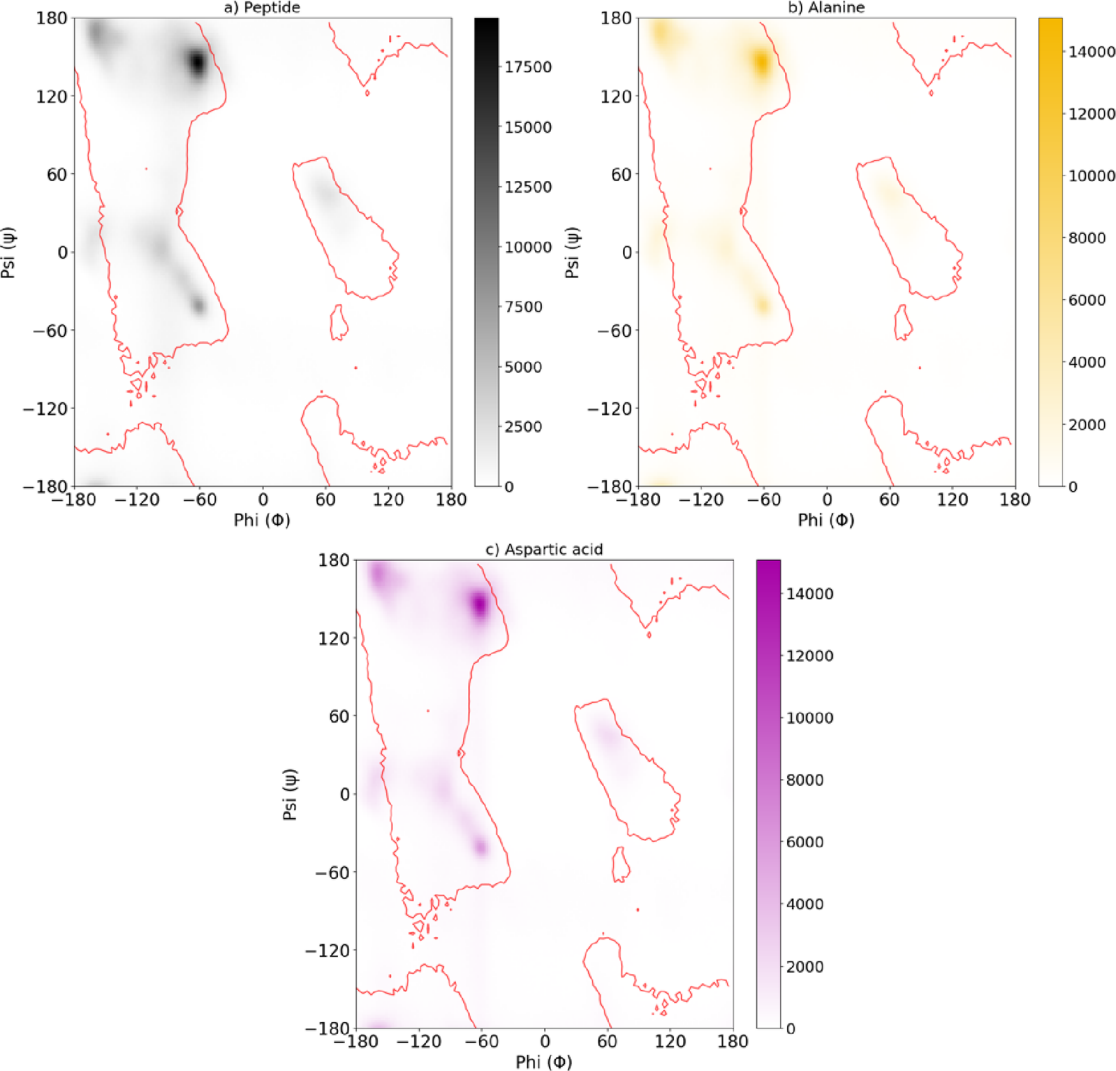
Ramachandran plots of the A_6_D peptide confined inside a CNT. (a) Global dihedral angle distribution for all residues of the peptide, (b) distribution for alanine residues, and (c) distribution for aspartic acid residues. The color intensity represents the population density of conformations sampled during the MD simulations. The contours (in red) indicate the most favorable regions of φ and ψ angles corresponding to α-helical and β-sheet conformations. The results reveal that the confined peptides predominantly sample α-helical regions, with alanine residues exhibiting a stronger helical preference near the CNT surface, while aspartic acid residues display broader sampling due to their polar and flexible nature. These conformational features highlight the balance between confinement-induced ordering and residue-specific flexibility within the CNT environment.

For the full peptide (Figure 4a), the distribution is highly concentrated within well-defined regions corresponding to typical α-helical and β-sheet conformations. The high density of these regions indicates that confinement within the CNT does not hinder the adoption of ordered secondary structures, suggesting conformationally stable and well-organized behavior. The alanine-specific plot (Figure 4b) shows a similar pattern, although with slightly lower density and moderate dispersion. This behavior reflects the small, nonpolar side chain of alanine, which imparts intermediate flexibility while still favoring α-helical and β-like regions. In contrast, the distribution for aspartic acid (Figure 4c) is more diffuse, with a reduced definition of the classical Ramachandran regions. This broad sampling arises from the charged carboxyl side chain of Asp, which introduces additional electrostatic and steric effects, enhancing conformational mobility. The larger occupation of intermediate regions suggests that Asp residues explore a wider conformational landscape, influenced by interactions with counterions, solvent molecules, and the confining CNT walls.

Overall, the Ramachandran analysis reveals a clear predominance of α-helical conformations over β-sheet ones. This preference can be attributed to the geometric compatibility between the α-helix and the cylindrical cavity of the CNT, which promotes the stabilization of compact, helical structures over extended β-sheet arrangements. The confinement therefore acts as an organizing factor, enhancing structural order while preserving local flexibility—a balance that may be crucial for maintaining mechanical resilience, cooperative hydrogen bonding, and functional stability of peptide–CNT hybrid materials in bioelectronic or nanocarrier applications.

Taken together, the analyses of hydrogen bonding, nonbonded interaction energies, and conformational distributions reveal a unified stabilization mechanism within the confined peptide–CNT system. Electrostatic solvation, dominated by aspartic acid–water interactions, prevents excessive aggregation and sustains the dynamic equilibrium of the peptides inside the nanotube. At the same time, dispersion forces drive hydrophobic residues, particularly alanine, toward the CNT surface, promoting adhesion and the formation of a semiorganized internal coating. This balance between solvation-driven flexibility and surface-induced ordering gives rise to a structurally coherent yet dynamically adaptable molecular arrangement. Such a synergistic interaction between electrostatic and van der Waals forces under confinement explains the coexistence of structural stability, conformational diversity, and high mobility, which are fundamental characteristics for the functional performance of peptide–CNT hybrid materials in solution.

## 4 Conclusions

This work provides an atomistic description of the structural, energetic, and conformational behavior of A_6_D peptides confined inside a CNT. The analyses of HB dynamics reveal that solvation overwhelmingly dominates over peptide–peptide aggregation, maintaining a high molecular mobility within the confined environment. Coulombic and van der Waals interaction profiles indicate that electrostatic stabilization is primarily governed by aspartic acid–water and peptide–ion interactions, while dispersion forces promote adhesion of alanine residues to the CNT walls. The combination of these interactions results in a semiordered peptide layer, where strong solvation and hydrophobic confinement coexist, ensuring both flexibility and structural coherence. Ramachandran analysis further demonstrates a predominant α-helical conformation that is geometrically compatible with the cylindrical CNT cavity, reflecting confinement-induced ordering.

Overall, these findings elucidate the molecular mechanisms that stabilize peptide–CNT hybrid systems under nanoscale confinement. The cooperative balance between solvation, electrostatic attraction, and dispersion-driven adhesion generates a stable, yet adaptable, structure that may serve as a foundation for biofunctional coatings and nanodevice design. The confined peptide assemblies described here open perspectives for applications in bioelectronic interfaces, selective molecular transport, nanofluidic membranes, and drug-delivery systems where structural tunability and charge regulation could be leveraged to control interfacial properties and molecular selectivity. Future extensions of this work could explore the effect of peptide sequence variation, CNT chirality, and external stimuli (e.g., electric fields, pH) to further tailor the structural and functional response of peptide-coated carbon nanostructures. Finally, the structural motifs identified here could guide future experimental efforts to engineer peptide-functionalized CNTs with tailored stability and solvation properties.

## Supplementary Material



## Notes

Time evolution of potential energy, temperature, simulation box volume, and hydrogen-bond numbers between peptides and between peptides and water during the MD simulations (Figure S1); comparative hydrogen-bond statistics, free-energy changes, hydrogen-bond lifetimes, and Coulombic and van der Waals interaction energies for peptide–peptide, peptide–water, residue–water, residue–ion, and residue–CNT interactions across three independently initialized simulated systems (Table S1) (PDF)

## References

[1] Mirzaei M., Meskinfam M., Yousefi M. (2012). Covalent hybridizations of carbon
nanotubes through peptide linkages: A density functional approach,
Comput. Theor. Chem..

[2] Talaei F., Farzad F., Yaghobi A. (2025). Molecular
insights into functionalized
carbon nanotubes for the adsorption of therapeutic peptides. Results in Materials.

[3] Mansouri A., Mahnam K. (2017). Designing new surfactant peptides
for binding to carbon
nanotubes via computational approaches. J. Mol.
Graph. Model..

[4] Barzegar A., Mansouri A., Azamat J. (2016). Molecular dynamics simulation of
non-covalent single-walled carbon nanotube functionalization with
surfactant peptides. J. Mol. Graph. Model..

[5] Wang S., Humphreys E. S., Chung S.-Y., Delduco D. F., Lustig S. R., Wang H., Parker K. N., Rizzo N. W., Subramoney S., Chiang Y.-M., Jagota A. (2003). Peptides with selective affinity
for carbon nanotubes. Nat. Mater..

[6] Sheikholeslam M., Pritzker M., Chen P. (2014). Hybrid peptide–carbon
nanotube
dispersions and hydrogels. Carbon N. Y..

[7] Wang J., Ouyang Z., Ren Z., Li J., Zhang P., Wei G., Su Z. (2015). Self-assembled peptide
nanofibers on graphene oxide
as a novel nanohybrid for biomimetic mineralization of hydroxyapatite. Carbon N. Y..

[8] Rozhin P., Adorinni S., Iglesias D., Mackiol T., Kralj S., Bisetto M., Abrami M., Grassi M., Bevilacqua M., Fornasiero P., Marchesan S. (2023). Nanocomposite
Hydrogels with Self-Assembling
Peptide-Functionalized Carbon Nanostructures. Chem.–Eur. J..

[9] Nepomuceno F. C., Kolář M. H. (2025). Sensitivity of peptide conformational
dynamics in carbon nanotubes to directional mechanical forces. Phys. Chem. Chem. Phys..

[10] Chen Q., Liang L., Zhang Z., Wang Q. (2021). Release of an Encapsulated
Peptide from Carbon Nanotubes Driven by Electric Fields: A Molecular
Dynamics Study. ACS Omega.

[11] Zhang Z.-S., Kang Y., Liang L.-J., Liu Y.-C., Wu T., Wang Q. (2014). Peptide encapsulation
regulated by the geometry of carbon nanotubes. Biomaterials.

[12] Chen Q., Zhou J., Sun R. (2024). Carbon Nanotube Loading Strategies
for Peptide Drugs: Insights from Molecular Dynamics Simulations. Langmuir.

[13] Meng F., Tang C., Wang B., Liu T., Zhu X., Miao P. (2016). Peptide and carbon nanotubes assisted detection of
apoptosis by square
wave voltammetry. Electrochim. Acta.

[14] Li W., Gao Y., Zhang J., Wang X., Yin F., Li Z., Zhang M. (2020). Universal
DNA detection realized by peptide based carbon nanotube
biosensors. Nanoscale Adv..

[15] Law S. S. Y., Kuzumoto M., Fujita S., Fujigaya T., Numata K. (2024). Carbon nanotubes
functionalized with α-aminoisobutyric acid-containing peptide
increase gene delivery efficiency in plant mitochondria. Polym. J..

[16] Sim D., Huang T., Kim S. S. (2023). Peptide-Functionalized Carbon Nanotube
Chemiresistors: The Effect of Nanotube Density on Gas Sensing. Sensors.

[17] Schrödinger, LLC. The PyMOL Molecular Graphics System. Schrödinger, LLC, 2015. https://www.pymol.org (accessed Feb 9, 2025).

[18] Humphrey W., Dalke A., Schulten K. (1996). VMD: Visual
molecular dynamics. J. Mol. Graph..

[19] Martínez L., Andrade R., Birgin E. G., Martínez J. M. (2009). PACKMOL:
A package for building initial configurations for molecular dynamics
simulations. J. Comput. Chem..

[20] Abraham M. J., Murtola T., Schulz R., Páll S., Smith J. C., Hess B., Lindah E. (2015). Gromacs: High
performance
molecular simulations through multi-level parallelism from laptops
to supercomputers. SoftwareX.

[21] Abraham, M. ; Alekseenko, A. ; Bergh, C. ; Blau, C. ; Briand, E. ; Doijade, M. ; Fleischmann, S. ; Gapsys, V. ; Garg, G. ; Gorelov, S. ; Gouaillardet, G. ; Gray, A. ; Irrgang, M. E. ; Jalalypour, F. ; Jordan, J. ; Junghans, C. ; Kanduri, P. ; Keller, S. ; Kutzner, C. ; Lemkul, J. A. ; Lundborg, M. ; Merz, P. ; Miletić, V. ; Morozov, D. ; Páll, S. ; Schulz, R. ; Shirts, M. ; Shvetsov, A. ; Soproni, B. ; van der Spoel, D. ; Turner, P. ; Uphoff, C. ; Villa, A. ; Wingbermühle, S. ; Zhmurov, A. ; Bauer, P. ; Hess, B. ; Lindahl, E. GROMACS 2023 Manual; Zenodo, 2023, DOI: 10.5281/zenodo.7588711.

[22] Hamley I. W., Hutchinson J., Kirkham S., Castelletto V., Kaur A., Reza M., Ruokolainen J. (2016). Nanosheet
Formation by an Anionic Surfactant-like Peptide and Modulation of
Self-Assembly through Ionic Complexation. Langmuir.

[23] Alves E. D., Oliveira L. B. A., Colherinhas G. (2019). Understanding
the stability of polypeptide
membranes in ionic liquids: A theoretical molecular dynamics study. New J. Chem..

[24] Brooks B. R., Brooks C. L., Mackerell A. D., Nilsson L., Petrella R. J., Roux B., Won Y., Archontis G., Bartels C., Boresch S., Caflisch A., Caves L., Cui Q., Dinner A. R., Feig M., Fischer S., Gao J., Hodoscek M., Im W., Kuczera K., Lazaridis T., Ma J., Ovchinnikov V., Paci E., Pastor R. W., Post C. B., Pu J. Z., Schaefer M., Tidor B., Venable R. M., Woodcock H. L., Wu X., Yang W., York D. M., Karplus M. (2009). CHARMM: The biomolecular simulation program. J. Comput. Chem..

[25] Best R. B., Zhu X., Shim J., Lopes P. E. M., Mittal J., Feig M., MacKerell A. D. (2012). Optimization of the additive CHARMM all-atom protein
force field targeting improved sampling of the backbone φ, ψ
and side-chain χ1 and χ2 Dihedral Angles. J. Chem. Theory Comput..

[26] Oliveira L. B. A., Colherinhas G. (2020). Can CHARMM36 atomic charges described correctly the
interaction between amino acid and water molecules by molecular dynamics
simulations?. J. Mol. Liq..

[27] Colherinhas G. (2021). Update of
CHARMM36’s atomic charges for aromatic amino acids in water
solution simulations and spectroscopy analysis via sequential molecular
dynamics and DFT calculations. J. Mol. Liq..

[28] Jorgensen W. L., Maxwell D. S., Tirado-Rives J. (1996). Development
and testing of the OPLS
all-atom force field on conformational energetics and properties of
organic liquids. J. Am. Chem. Soc..

[29] Jorgensen W. L., Chandrasekhar J., Madura J. D., Impey R. W., Klein M. L. (1983). Comparison
of simple potential functions for simulating liquid water. J. Chem. Phys..

[30] Parrinello M., Rahman A. (1981). Polymorphic transitions in single crystals: A new molecular
dynamics method. J. Appl. Phys..

[31] Bussi G., Donadio D., Parrinello M. (2007). Canonical
sampling through velocity
rescaling. J. Chem. Phys..

[32] Darden T., York D., Pedersen L. (1993). Particle mesh
Ewald: An N·log­(N)
method for Ewald sums in large systems. J. Chem.
Phys..

[33] Hess B., Bekker H., Berendsen H. J. C., Fraaije J. G. E. M. (1997). LINCS: A Linear
Constraint Solver for molecular simulations. J. Comput. Chem..

[34] Luzar A. (2000). Resolving
the hydrogen bond dynamics conundrum. J. Chem.
Phys..

[35] Luzar A., Chandler D. (1996). Hydrogen-bond kinetics
in liquid water. Nature.

[36] Van
Der Spoel D., Van Maaren P. J., Larsson P., Tîmneanu N. (2006). Thermodynamics
of hydrogen bonding in hydrophilic and hydrophobic media. J. Phys. Chem. B.

[37] Ramachandran G. N., Ramakrishnan C., Sasisekharan V. (1963). Stereochemistry of polypeptide chain
configurations. J. Mol. Biol..

[38] Sam A., Kannam S.K., Hartkamp R., Sathian S.P. (2017). Water flow in carbon
nanotubes: The effect of tube flexibility and thermostat. J. Chem. Phys..

