# Supplementary material for: Peptide–Carbon Nanotube Hybrids under Confinement: Structure and Stability from Atomistic Simulations
Source: ACS Omega. 2026 Feb 11;11(7):12602–11. doi: 10.1021/acsomega.5c12748 (PMC12947170; doi:10.1021/acsomega.5c12748)
Supplement: Supplementary file 1 [file ao5c12748_si_001.pdf]

SUPPORTING INFORMATION

Peptide–Carbon Nanotube Hybrids Under Confinement: Structure and Stability from Atomistic Simulations

Karinna Mendanha[1] and Guilherme Colherinhas[1]*

[1] Instituto de Física, Universidade Federal de Goiás, 74690-900, Goiânia, GO, Brazil.
[*] corresponding author: gcolherinhas@ufg.br or gcolherinhas@gmail.com (GC)

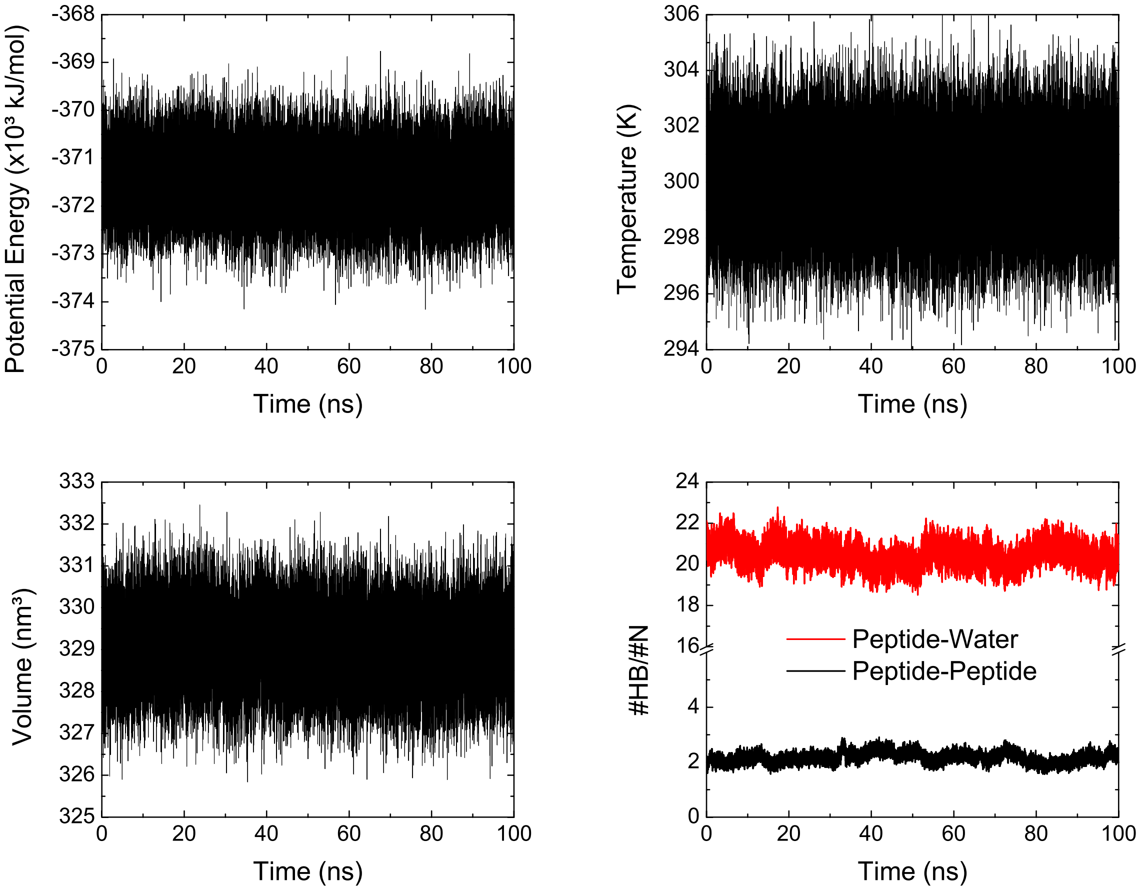

Figure S1: *Time evolution of the Potential Energy of the system (in x10³ kJ/mol), Temperature of the system (in K), Volume of simulation box (in nm³), and number of HB between peptides and between peptides and water molecules (per peptides) during the molecular dynamics simulation.*

Table S1: *Comparative results among the three similar simulated systems. Systems 02 and 03 were simulated for comparison with the results obtained for System 01. All systems have the same composition and follow the same MD simulation protocol, differing only in their initial configurations. The results highlight: the average number of hydrogen bonds (HBs), ΔG (kJ mol^{-1}), and HB lifetimes (ps) obtained from the Luzar–Chandler theory; as well as the average Coulomb (E_C) and van der Waals (E_{LJ}) interaction energies per residue (kJ mol^{-1}·N^{-1}) and the corresponding root-mean-square deviations (RMSD) for alanine and aspartic acid residues interacting with water, ions, and the carbon nanotube (CNT).*

		**# HB**	**ΔG**	**HB-Lifetime**
**Peptide-Peptide**	**System 01**	2.17 ± 0.19 per peptide	24.57 kJ/mol	3247.15 ps
	**System 02**	2.13 ± 0.24 per peptide	24.62 kJ/mol	3304.84 ps
	**System 03**	2.25 ± 0.28 per peptide	24.12 kJ/mol	2702.77 ps
	**Average**	2.18 ± 0.24 per peptide	24.44 kJ/mol	3084.92 ps
**Peptide-Water**	**System 01**	20.47 ± 0.53 per peptide	14.34 kJ/mol	52.87 ps
	**System 02**	20.56 ± 0.76 per peptide	13.97 kJ/mol	45.08 ps
	**System 03**	20.33 ± 0.82 per peptide	14.02 kJ/mol	45.98 ps
	**Average**	20.45 ± 0.70 per peptide	14.11 kJ/mol	47.98 ps
**Alanine-Water**	**System 01**	1.64 ± 0.06 per alanine	15.15 kJ/mol	72.67 ps
	**System 02**	1.67 ± 0.10 per alanine	14.45 kJ/mol	54.85 ps
	**System 03**	1.65 ± 0.08 per alanine	14.67 kJ/mol	59.72 ps
	**Average**	1.65 ± 0.08 per alanine	14.76 kJ/mol	62.41 ps
**Aspartic Acid-Water**	**System 01**	10.61 ± 0.34 per aspartic acid	13.54 kJ/mol	37.88 ps
	**System 02**	10.55 ± 0.35 per aspartic acid	13.54 kJ/mol	37.92 ps
	**System 03**	10.44 ± 0.43 per aspartic acid	13.42 kJ/mol	36.12 ps
	**Average**	10.53 ± 0.37 per aspartic acid	13.50 kJ/mol	37.31 ps

	**System 01**	**System 02**	**System 03**	**Average**
**Coulomb Energy (E_C)**	**E_C/N (kJ/mol)**	**E_C/N (kJ/mol)**	**E_C/N (kJ/mol)**	**E_C/N (kJ/mol)**
**Alanine-Water**	-50.63 ± 2.66	-51.79 ± 3.54	-50.88 ± 3.17	-51.10 ± 3.12
**Aspartic Acid-Water**	-593.44 ± 18.05	-594.72 ± 20.10	-587.18 ± 23.99	-591.78 ± 20.71
**Alanine-Ions**	-0.44 ± 0.75	-0.21 ± 0.85	0.07 ± 0.80	-0.19 ± 0.80
**Aspartic Acid-Ions**	-127.10 ± 15.50	-135.88 ± 17.79	-142.29 ± 20.74	-135.09 ± 18.01
**Alanine-CNT**	0	0	0	0
**Aspartic Acid-CNT**	0	0	0	0
**Van der Waals Energy (E_{LJ})**	**E_{LJ}/N (kJ/mol)**	**E_{LJ}/N (kJ/mol)**	**E_{LJ}/N (kJ/mol)**	**E_{LJ}/N (kJ/mol)**
**Alanine-Water**	-6.86 ± 0.58	-6.95 ± 0.61	-7.30 ± 0.59	-7.04 ± 0.59
**Aspartic Acid-Water**	28.89 ± 3.00	29.07 ± 3.03	28.03 ± 3.14	28.66 ± 3.06
**Alanine-Ions**	0.03 ± 0.08	0.03 ± 0.09	0.02 ± 0.42	0.03 ± 0.20
**Aspartic Acid-Ions**	6.22 ± 1.42	6.70 ± 1.52	7.20 ± 1.67	6.71 ± 1.54
**Alanine-CNT**	-17.35 ± 0.80	-16.15 ± 1.05	-15.74 ± 0.85	-16.41 ± 0.90
**Aspartic Acid-CNT**	-3.45 ± 0.46	-3.67 ± 0.46	-3.66 ± 0.52	-3.59 ± 0.48
